# Performance and Regeneration Evaluation of rGO Filter Materials During Ultrasonic Cleaning with Different Cleaning Solutions

**DOI:** 10.3390/ma19030566

**Published:** 2026-02-01

**Authors:** Tao Yu, Wenjun Leng, Xin Zhang, Qing Liu

**Affiliations:** 1 School of Energy and Power Engineering, Beihang University, Beijing 100191, China; 2 Wuhan Second Ship Design and Research Institute, Wuhan 430205, China; 3 College of Architecture and Energy Engineering, Wenzhou University of Technology, Wenzhou 325000, China; 4 School of Resources Engineering, Xi′an University of Architecture and Technology, Xi’an 710055, China; qingliu@xauat.edu.cn

**Keywords:** rGO filter material, water, lemon acid, cleaning agent, filtration performance, regeneration evaluation

## Abstract

The regeneration of air filter materials can extend the service life of filters, and also reduce resource waste and air pollution caused by replacements, which directly lower carbon emissions. This paper focuses on reduced graphene oxide (rGO) filter materials, investigating the effects of ultrasonic cleaning utilizing water, lemon acid, and a cleaning agent. Regeneration performances were also tested and discussed and analyzed. Results show the synergistic effect of the cleaning agent and ultrasonic cleaning yields the most optimal regeneration performance. Compared to the water and lemon acid, filtration efficiency of rGO materials for PM_10_, PM_2.5_, and PM_1.0_ increased by 2.0%~12.15% and 0.42%~7.13%, 0.04%~5.67% and 0.03%~2.35%, and 0.02%~3.47% and 0.16%~2.02%, respectively. Filtration efficiency recovery rates for PM_10_, PM_2.5_, and PM_1.0_ using the cleaning agent exceeded 70%. Counting filtration efficiency exhibited significant changes for particle sizes from 0.265 to 1.0 μm. The resistance after water cleaning was higher than that of cleaning agent cleaning and lemon acid cleaning. After 10 cleaning cycles, the cleaning agent exhibited QF values that were 0.0012 Pa^−1^, 0.0003 Pa^−1^, and 0.0001 Pa^−1^ higher for PM_10_, PM_2.5_, and PM_1.0_, respectively, compared to the water, and 0.0007 Pa^−1^, 0.0001 Pa^−1^, and 0.0001 Pa^−1^ higher compared to the lemon acid. It provides data references for the efficient regeneration of rGO materials and promotes the green application of air filter materials.

## 1. Introduction

Atmospheric pollution has always been a globally concerning environmental issue. Particles can enter the human body through the respiratory tract, causing various diseases and even death [1,2]. As one of the core means of controlling atmospheric particulate pollution, air filter technology relies on the research and application of filter materials. Conventional filter materials have drawbacks, such as their low particle capture efficiency, easy clogging, and difficult regeneration [3]. Reduced graphene oxide (rGO), with its unique structure, can physically intercept particles of different sizes. At the same time, oxygen-containing functional groups can enhance the particle capture ability through electrostatic adsorption and chemical adsorption, demonstrating significant advantages in the field of efficient filtration [4,5]. However, rGO filter materials still face the problem of particle accumulation on the surface and within the pores, which may increase the filtration resistance and decrease the filtration efficiency. Simultaneously increasing system energy consumption [6] and directly replacing materials will lead to resource waste and cost escalations. Therefore, the regeneration of rGO filter materials is currently pressing research topics.

At present, the regeneration methods of rGO filter materials mainly include water cleaning, thermal regeneration, chemical cleaning, and ultrasonic cleaning [7,8,9,10,11,12,13,14,15]. Domestic and foreign scholars have conducted preliminary explorations on different regeneration methods. Among them, the water cleaning method has become a focus of early research due to its simple operation and low cost. The relevant literature [7] demonstrates that air filters composed of reduced graphene oxide can be cleaned twice using the water cleaning method, and after more than two cleaning cycles, they cannot no longer achieve the required value of 85%. The particles washed from the inside of the fibers under the water flow action may also have an impact on the fiber structure. The limitation of water cleaning lies in the strong electrostatic adsorption between particulate matter and functional groups of rGO [8], which cannot be overcome by physical flushing alone. In addition, relevant studies have confirmed that rinsing with clean water can remove some particles on the surface of the material through the water flow action. However, for fine particles embedded in the interlayer pores of rGO, the permeability and stripping ability of water cleaning methods are limited, which can easily lead to particle residue. Moreover, repeated water cleaning can damage the integrity of the layered structure of the rGO material, resulting in an irreversible decrease in filtration efficiency [9].

To address the limitations of regeneration via water cleaning methods, cleaning media such as surfactants and acid–base solutions are utilized to weaken the adsorption force between particles and rGO materials through chemical reactions, assisting in the desorption of particles. Existing research has shown that cleaning solutions such as sodium dodecylbenzenesulfonate (SDBS) and lemon acid can effectively improve the removal rate of particulate matter [10]. However, traditional chemical cleaning relies on soaking or stirring methods, and cleaning efficiency is limited by the mass transfer rate. Moreover, excessive chemical reagents are prone to residue on the rGO surface, causing secondary pollution or functional group modification, which in turn damages the material’s filtration performance [11].

In contrast, ultrasonic cleaning, relying on the micro-jet and shock waves generated by cavitation effects, can penetrate deeply into the microporous structure of rGO materials with low mechanical damage, causing strong particle detachment. At the same time, the vibration effect of ultrasonic waves can enhance the contact between the cleaning solution and the material surface, combining the synergistic advantages of physical flushing with chemical action [12]. Previous studies have found that under repeated dust containment conditions, non-woven fabrics can also restore their filtration performance to over 85% of the clean filter material when using ultrasonic cleaning methods for PM_10_ (nine times), PM_2.5_ (six times), and PM_1.0_ (seven times) [13]. However, the relevant literature suggests that using chemical solvents for cleaning not only reduces the hydrophilicity of the material surface but also makes it easier for particles to adhere during secondary filtration [14]. At the same time, the acidity and alkalinity can corrode fibers, which may decrease the filtration efficiency [15].

Although the advantages of washing with ultrasonic water have been verified, the research on the synergistic regeneration of rGO filter materials using an “ultrasonic + cleaning solution” is still in its infancy. At present, researchers mainly focus on the ultrasonic regeneration effect of a single cleaning medium (water), and systematic comparative studies that focus on the mechanism of action of different types of cleaning solutions (surfactant solution, acid–base solution, air conditioning cleaning solution, etc.) in the ultrasonic field, their effects on the microstructure and surface properties of rGO materials, and the filtration performance of regenerated materials (efficiency, resistance, dust-holding capacity, and quality factor) have not been carried out [16,17]. Moreover, there is a lack of in-depth data regarding the long-term effects of cleaning solutions on the structure of rGO materials, as well as the stability of their performance after multiple regenerations. These issues directly relate to the practical application value of ultrasonic cleaning technology in rGO filter material regeneration, representing gaps in this research field. In addition, the existing work on regeneration technology is mostly focused on traditional fiber filter materials (such as polypropylene and glass fiber) [18], and there is relatively insufficient research on the regeneration characteristics of new carbon-based filter materials such as rGO. There is no systematic report on the effect of ultrasonic coupling cleaning solutions on the effect of rGO filter materials, and the research on the retention and resistance characteristics of multi-particle size combinations after rGO cleaning is seriously insufficient.

This paper focuses on the synergistic regeneration effect of differences for cleaning solutions on rGO filter materials during ultrasonic cleaning. Through a comprehensive evaluation of filtration efficiency, resistance, and quality factors, the regeneration performance of water, lemon acid, and a cleaning agent is systematically compared. The provision of a theoretical basis and technical support for the efficient and stable regeneration of rGO filter materials also has great significance for promoting the low-cost application of air filter materials.

## 2. Methods

### 2.1. Parameters

Filtration efficiency is shown in Equation (1) [7].

(1) $$\eta =\frac{C_{1}-C_{2}}{C_{1}}\times 100\%$$

where *η* is filtration efficiency (%); *C*_1_ is the concentration of particulate matter before filtration (μg/m^3^); and *C*_2_ is the concentration of particulate matter after filtration (μg/m^3^).

Filtration resistance is shown in Equation (2) [7].

(2) $$∆P=P_{2}-P_{1}$$

where *P*_1_ is the pressure before filtration (Pa), and *P*_2_ is the pressure after filtration (Pa).

Filtration efficiency recovery rates are shown in Equation (3) [19].

(3) $$ERR=\frac{\eta_{W}}{\eta_{WO}}\times 100\%$$

where ERR is the recovery rate of filtration efficiency, %; $\eta_{WO}$ is the initial filtration efficiency, %; and $\eta_{W}$ is the filtration efficiency after cleaning, %.

Quality factor (*QF*) is shown in Equation (4) [9,20].

(4) $$QF=\frac{-ln\left(1-\eta \right)}{\Delta p}$$

where *η* is the filtration efficiency, %, and ∆P is the filtration resistance, Pa.

Cleanliness is shown in Equation (5) [7].

(5) $$\eta =(1-\frac{m}{M})\times 100\%$$

where *η* is cleanliness (%); *m* is the mass of residual dust after cleaning (g); and *M* is the initial mass of the clean filter material (g).

### 2.2. Experimental Content

**Figure 1:**
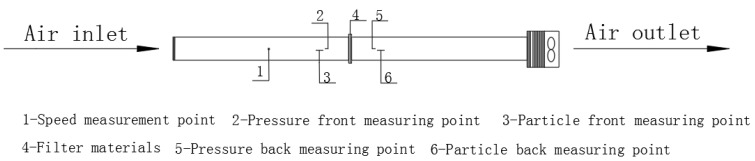
Experimental setup [13].

The testing platform used in the experiment is shown in Figure 1 [13]. The concentrations of particles before and after the air filter was measured using GRIMM1.109 Portable Aerosol Spectrometer—the specific testing locations of which were measuring points 3 and 6, shown in Figure 1. The device was supplied by Beijing Saak-Mar Environmental Instrument Ltd., Beijing, China. The upper limit of concentration that could be counted was 2,000,000 P/L. The measurement range was 0.1~100,000 μg/m^3^, with a particle size range of 0.25–32 μm, a repetition rate of 5%, and a sampling frequency of 1 Hz. This instrument uses optical scattering principles to detect particulate matter, which is suitable for the real-time monitoring of high-concentration and rapidly changing particulate matter in environments. The instruments underwent systematic calibration before and during the test. Nominal sampling flow rate: 1.2 L/min (factory-set for GRIMM1.109 Portable Aerosol Spectrometer). Flow rate stability: ±2% of nominal value. Every 3 days, a zero-point calibration was performed for the Grimm1.109. The instrument was placed in a clean room (Class 100) for 30 min, and the zero-point offset was adjusted to ≤0.01 μg/m^3^ to eliminate background interference. After each day’s sampling, the inlet nozzle of the Grimm1.109 was cleaned with compressed air (pressure: 0.3 MPa) to remove particulate matter residues and avoid blockages. Battery levels of both instruments were checked daily, and spare batteries were prepared to avoid power outages during sampling. During the experiment, the particle concentration upstream of the filter (C_1_) was maintained below 500,000 P/L (far below the instrument’s upper limit), ensuring the probability of coincidence was <0.5% (calculated via the instrument’s built-in coincidence model: P = 0.5 × C × t, where C = concentration, and t = detection time window = 1 μs). For each measurement, 5 min average concentrations were recorded (instead of shorter intervals) to smooth out random coincidence events.

The filtration resistance was measured using a HD2114P.0 Portable Micromanometer, supplied by DeltaOHM Co., Ltd., Selvazzano (PD), Italy, with an accuracy of ±(2% reading + 0.1 m/s). Pressure range was ±0.4% F.S. Velocity was measured using HD37AB1347 Indoor Air Quality Monitor, supplied by DeltaOHM Co., Ltd., Selvazzano (PD), Italy, accuracy range ± 3%. The HD37AB1347 monitor was calibrated against a primary standard Pitot tube (accuracy ±1%) before the experiment. Calibration points: 0.2, 0.5, 0.8, and 1.0 m/s; calibration certificate traceable to NIST. Zero-point calibration for velocity: conducted in a Class 100 cleanroom (air velocity < 0.01 m/s) before each test day. Face velocity uncertainty: ±3% (instrument accuracy) + ±2% (spatial variation, n = 5 measurements) = ±5% (combined standard uncertainty). Flow rate uncertainty: propagated from velocity (±5%) and area (±1%, from dimensional measurement) = ±5.1% (via root-sum-square method). An XCS-101-0BS drying oven was used for drying; temperature ranged from room temperature to 300 °C. The ultrasonic cleaning machine (KQ-500DE, Kunshan Ultrasonic Instrument, Kunshan, China) has an adjustable power range of 0–500 W.

Filtration performance measurements were conducted in a controlled laboratory environment at the Xi′an University of Architecture and Technology (Xi’an, China), under ambient temperature (26.8 °C~41.4 °C) and relative humidity (42.7%~62.3%), such as all filtration efficiency, resistance, and quality factor measurements. Environmental parameters were recorded every 10 min during testing using a calibrated thermo-hygrometer (accuracy: T ±0.1 °C, RH ± 1%). Dimensions: 12 cm × 12 cm (outer size), with an effective filtration area of 10 cm × 10 cm (inner active area). Quantity: 60 specimens total (10 cleaning cycles × 3 cleaning solutions × 2 specimens per group). We used the average concentration before and after the test for 5 min for calculation and analysis to reduce experimental errors during the period of August–October 2024. This operation fully ensured the repeatability and representativeness of the experimental data, effectively reducing the deviation of a single test.

In this study, L-ascorbic acid was used for multiple reduction treatments in the preparation of rGO, achieving a significant reduction in graphene oxide. This process can fully remove active oxygen-containing groups, significantly reducing the active sites on the material surface that react with citric acid. Meanwhile, the chemical properties of rGO after the reduction are more stable, which can effectively avoid further reductions or side reactions caused by the citric acid. The porosity (%) of rGO filter is 94.62 ± 0.03, and the filling rate is 5.38 ± 0.03. All parameters are measured for the original (uncleaned) rGO filter material, with 3 replicates per measurement. The low Reynolds number (Re < 0.01) confirms that fluid flow through the filter pores is laminar, which is consistent with typical air filtration conditions.

Based on previous results [21], dust samples from air conditioning systems were used for dust loading experiments, and collected from residential HVAC filters (Xi’an, China) and pooled to ensure homogeneity. Pretreatment steps: Sieving: Passed through a 150 μm stainless steel sieve to remove large debris (e.g., hair, fibers). Drying: Dried at 60 °C for 4 h to remove moisture (moisture content < 2% verified by mass balance). Homogenization: Mixed using a rotary mixer (1000 rpm, 30 min) to ensure uniform particle distribution. Storage: Sealed in polyethylene containers at 25 °C, <30% RH to prevent agglomeration. The proportion of particle sizes of 0.3–2.5 μm in the dust sample is 95.5%, the proportion of particle sizes of 0.5–2.5 μm is 49.3%, and the proportion of particle sizes of 0.5–5.0 μm is 55.7% [21]. The dust samples contained a large number of small particles, and it is consistent with the actual situation where small particles dominate the atmosphere. Therefore, using dust samples as a dust source for testing has practical engineering significance. Target Loading: 5.0 ± 0.2 mg/cm^2^ (consistent across all rGO filter samples). Dust was aerosolized using a constant-output nebulizer (flow rate: 5 L/min) and directed onto rGO filters until the target mass was achieved. Post-loading mass was measured using a microbalance (accuracy: ±0.01 mg); filters were weighed before and after loading (mass difference = dust loading). PSD and mass loading data were recorded for each batch of dust (n = 3 batches); relative standard deviation (RSD) of D50 and mass.

Three experimental groups were designed based on cleaning solutions, with identical ultrasonic parameters for all groups: Water group was urban tap water. Lemon acid group: the lemon acid was prepared by China National Pharmaceutical Group Chemical Reagent Co., Ltd., Shanghai, China, (analytical grade, AR) in a 0.1 mol/L lemon acid solution. Cleaning agent group: The selected cleaning agent was the Dili brand 827 M air conditioner cleaning agent, diluted with deionized water to a concentration of 1% according to instructions. Based on the manufacturer’s technical sheet and product manual for Dili brand 827 M air conditioner cleaning agent, supplied by Shenzhen Dili Chemical Co., Ltd., Shenzhen, China, the key functional components are summarized as follows: surfactant (core), key ingredients: anionic surfactant (sodium alkylbenzene sulfonate, ~5–8%); chelating agent, key ingredients: sodium gluconate (~1–2%); corrosion inhibitor, key ingredients: sodium silicate (~0.5–1%); cosolvent, key ingredients: ethanol (~2–3%); pH adjuster, key ingredients: sodium hydroxide (~0.3–0.5%), based on a review of the product manual and technical parameters provided by the manufacturer, supplemented by a small amount of cosolvents (ethanol) and corrosion inhibitors (sodium silicate). Its formula is suitable for cleaning dust and oil stains on the surface of air conditioning filters and heat exchangers. Ten consecutive cleaning-regeneration cycles per group took place. Each cycle included (1) dust loading (HVAC system dust, target mass loading 5.0 mg/cm^2^), (2) ultrasonic cleaning (set parameters), (3) rinsing with deionized water, (4) drying (60 °C for 2 h), and (5) performance testing (filtration efficiency, resistance, etc.).

For rinsing: Pre-rinsing: Immediately after ultrasonic cleaning (120 W, 35 s), transfer the rGO filter material to a clean beaker filled with deionized water (resistivity ≥ 18.2 MΩ·cm, 23 °C ± 2 °C). Agitation rinsing: Place the beaker on a magnetic stirrer (500 rpm) for 2 min to remove surface-adhered cleaning solution. Repeat rinsing: Replace the deionized water and repeat the agitation rinsing step 3 times (total rinsing time: 6 min) to ensure thorough removal of residual reagents. Final rinse: Rinse the material with a gentle stream of deionized water (flow rate: 50 mL/min) for 30 s, directing the stream evenly across the filter surface and edges.

For drying: Pre-drying: Blot the rinsed filter material with lint-free filter paper to remove excess surface water (avoid pressing to prevent structural deformation). Oven drying: Place the material in an XCS-101-0BS drying oven preheated to 60 °C ± 2 °C, supplied by Shangcheng Instrument Manufacturing Co., Ltd., Shaoxing, China. Position the material on a wire mesh rack (not in direct contact with the oven wall) to ensure uniform air circulation. Drying duration: Maintain at 60 °C for 2 h; extend to 3 h if the material still feels damp (verified by constant mass). Cooling: After drying, transfer the material to a desiccator (relative humidity ≤ 10%) and cool to room temperature (23 °C ± 2 °C) for 30 min before performance testing.

**Figure 2:**
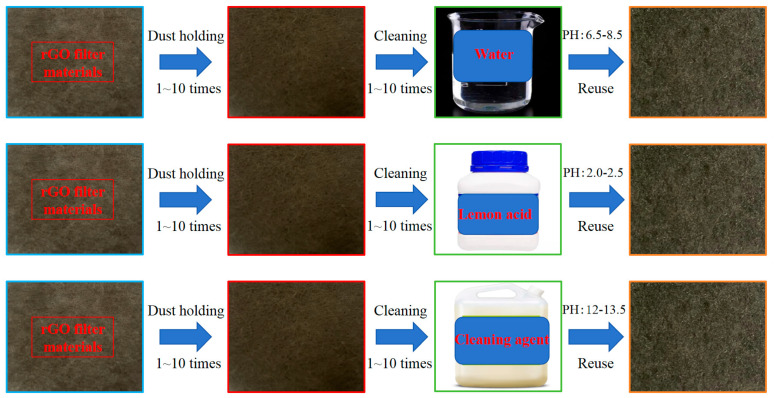
Processes using different cleaning solutions.

For each specimen, performance parameters (efficiency, Delta P, QF) were measured 3 times consecutively, with 5 min stabilization between measurements. Each group included 2 parallel specimens, and 3 independent experimental groups were conducted (total n = 6 measurements per data point). The average of 3 intra-specimen measurements was used as the individual specimen value, and SD was calculated across 6 inter-specimen/group measurements. Ultrasonic cleaning process was at room temperature (temperature 21 °C~25 °C, relative humidity 45%~55%), such as cleaning of dust-loaded rGO filters (1–10 cycles), and the process is shown in Figure 2.

The following table presents key physicochemical properties of the three cleaning solutions, measured at the experimental temperature (consistent with the ultrasonic cleaning environment). For water, PH: 7.2 ± 0.01, conductivity: 386 μS/cm, ±5%; lemon acid, 2.3 ± 0.01, conductivity: 1245 μS/cm, ±5%; cleaning agent 12.8 ± 0.01, conductivity: 892 μS/cm, ±5%. All measurements were repeated 3 times, and the average is reported with the corresponding relative standard deviation (RSD). The surface tension of the cleaning agent is lower than that of water and citric acid, which may contribute to its ability to penetrate rGO micropores and reduce capillary damage. The high conductivity of the citric acid solution is attributed to its dissociation into hydrogen ions and citrate ions, while the cleaning agent’s conductivity is derived from ionic surfactants and corrosion inhibitors.

### 2.3. Filtration System Parameters and Flow Rate Calculation

The detailed system-related calculations are shown in Table 1.

The effective filter area was determined by measuring the inner diameter of the holder’s flow channel (10 cm × 10 cm) to exclude non-flow regions.

Leakage was minimized by pre-tightening the holder’s clamping bolts to 5 N·m torque and verifying with a particle counter (Grimm1.109) by comparing upstream/downstream concentrations without the filter (leakage ≤ 0.5% was considered acceptable).

A linear mixed-effects (LME) model was selected to account for, random variation between specimens (each specimen was measured repeatedly across 10 cycles, creating nested data), and fixed effects of key experimental factors (cleaning solution, cycle number, and particle class) and their interactions. This model is robust to repeated-measures data and avoids overestimating significance compared to standard ANOVA.

## 3. Results and Discussion

### 3.1. Ultrasonic Cleaning Parameters

**Figure 3:**
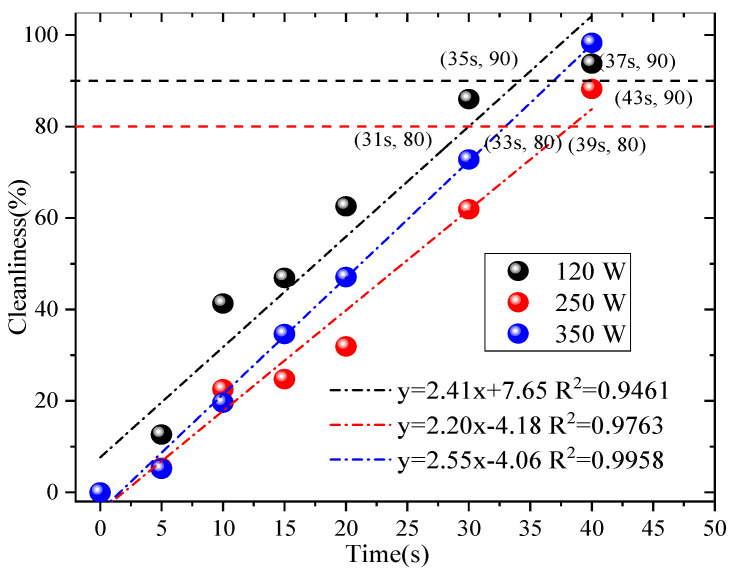
Related factors.

In-depth research was conducted on the factors of ultrasonic cleaning with reference to the relevant literature [9]. Tap water was used for ultrasonic cleaning, and only the power and cleaning time of the cleaning equipment were considered to affect the ultrasonic cleaning effect. The selected ultrasonic power range (120 W, 250 W, and 350 W) and time range (5 s, 10 s, 15 s, 20 s, 30 s, and 40 s) were determined based on three key considerations: Material compatibility: rGO filter materials have a layered structure that is sensitive to excessive mechanical force [9,21]. Powers above 350 W were avoided to prevent cavitation-induced damage to the rGO’s π-π stacking and interlayer hydrogen bonding, which would irreversibly reduce the filtration efficiency. Cleaning effectiveness: Powers below 120 W were excluded because preliminary trials align with [9] (Wei et al., 2019), which demonstrates that ultrasonic power < 120 W generates insufficient cavitation intensity to penetrate rGO micropores and strip embedded fine particles. Practical feasibility: Time durations exceeding 40 s were rejected to avoid unnecessary energy consumption and potential fiber swelling (from prolonged liquid contact), while durations shorter than 5 s were too brief to achieve meaningful particle desorption [12,21]. Controlling a single variable (40 KHZ), the designed ultrasonic power was 120 W, 250 W, and 350 W. The ultrasonic time was 5 s, 10 s, 15 s, 20 s, 30 s, and 40 s. We utilized Formula (5) to determine the cleanliness of different ultrasonic powers and ultrasonic times and analyze the relevant factors, as shown in Figure 3.

From Figure 3, the use of the ultrasonic cleaning method has an effect on the rGO filter material. When reaching 80% of the original cleanliness at different powers, 120 W, 250 W, and 350 W, the corresponding times are 31 s, 39 s, and 33 s. If reaching 90% of the original cleanliness at different powers, the corresponding times are 35 s, 43 s, and 37 s, respectively. Therefore, based on the 90% the original cleanliness—the economic costs and operability factors are taken into account, such as ultrasound electricity and water consumption—the best effect is achieved when using an ultrasound power of 120 W and a cleaning time of 35 s. This is consistent with the results presented in reference [21]. Subsequent experiments were conducted using ultrasonic parameters with the best cleaning effect. SEM images have been provided in previously published results [13], which reveal that some fibers exhibited some wear or damage after 10 washes. The reason for this may be that the sharp edges of the particles damaged their fiber structure when separated from the fibers; however, they still maintained good filtration efficiency, which verifies the applicability of this cleaning process.

### 3.2. The Influence of Filtration Efficiency

**Figure 4:**
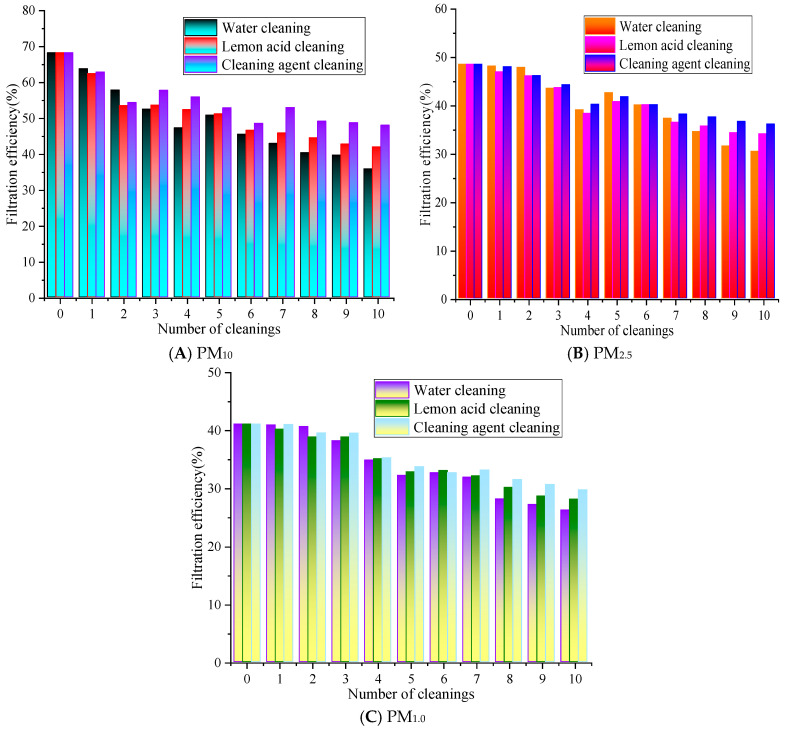
The influence of cleaning times on the filtration efficiency.

Based on previous results, experiments were conducted at a filtration velocity of 0.8 m/s [22]. This filtration velocity not only meets the common application conditions of air filter materials but also ensures the stability and discrimination of filtering efficiency and resistance detection data. The changes in the filtration performance are shown in Figure 4.

In Figure 4, as the number of cleaning cycles increases, the overall filtration performance of the rGO filter material exhibits a gradually decreasing trend. After the ultrasonic cleaning with water for 1 to 10 cleaning cycles, the filtration efficiency range is from 35.83% to 63.75%, 30.51% to 48.19%, and 26.26% to 40.98% for PM_10_, PM_2.5_, and PM_1.0_, respectively, while with lemon acid for 1 to 10 cleaning cycles, the filtration efficiency for PM_10_, PM_2.5_, and PM_1.0_ ranges from 41.95% to 62.41%, 34.16% to 47.02%, and 28.11% to 40.23%, respectively. The filtration efficiency for PM_10_, PM_2.5_, and PM_1.0_ with cleaning agents ranges from 47.98% to 62.83%, 36.18% to 48.07%, and 29.73% to 41.05%, respectively. Overall, the efficiency of cleaning with the agent/lemon acid is higher than that of water. The filtration efficiency of PM_10_, PM_2.5_, and PM_1.0_ is 2.0% to 12.15% and 0.42% to 7.13%, 0.04% to 5.67% and 0.03% to 2.35%, 0.02% to 3.47% and 0.16% to 2.02% higher than that of the water and lemon acid, respectively.

After 10 cleaning cycles with water, the filtration efficiency of PM_10_ decreased the most, by 32.44%, while the cleaning agent resulted in the smallest decrease of 20.29%. The PM_2.5_ efficiency with the lemon acid method is slightly higher than that of the cleaning agents, and the water cleaning method is always at the lowest level. This is because PM_2.5_’s particle size is smaller, making it easier to penetrate into the pores of rGO filter materials, and polar pollutants such as carbonates often adhere to its surface. Lemon acid has a weak acidic chelating effect [23], which may destroy the electrostatic and chemical adsorption forces between particles and rGO fibers. At the same time, lemon acid molecules exhibit strong polarity and can better penetrate into the micropores of the material, swelling and stripping the embedded PM_2.5_. The core component of air conditioning cleaning agents is surfactants, which mainly reduce interfacial tension and disperse non-polar pollutants [24]. Their effect on polar PM_2.5_ and tightly bound pollutants is weaker than the chelating and dissolving effect of citric acid, so their cleaning efficiency for PM_2.5_ is slightly inferior. However, water-based methods may only rely on physical flushing and ultrasonic cavitation effects to exert effects [21], lacking chemical reaction sites. The use of simple physical actions makes it difficult to effectively overcome these adsorption forces; thus, they cannot completely remove PM_2.5_ inside pores. Therefore, the clean water’s cleaning effect for PM_2.5_ is significantly weaker than that of chemical cleaning media.

The overall filtration efficiency of PM_1.0_ is lower than that of PM_10_ and PM_2.5_, and the rate of decrease with the number of cleaning cycles is faster. The cleaning agent has the best efficiency retention effect on PM_1.0_, followed by lemon acid. The efficiency of PM_1.0_ cleaned with water significantly decreases after five cleaning cycles, and after ten cleaning cycles, the efficiency is close to 50% of the initial value. As can be observed for all cleaning solutions, the greater the filtration efficiency decrease with the number of cleaning cycles. This is because water maybe only physically flush surface particles [21], which results in the worst protection of the filter material’s performance. Lemon acid can dissolve the alkaline components in particles through acid–base reactions, while non-ionic surfactants in cleaning agents can reduce the interfacial tension between particles and fibers, thereby effectively removing stubborn particles inside fiber pores, ultimately alleviating material blockages and restoring the filtration performance.

### 3.3. The Influence of Filtration Efficiency Recovery Rate

**Figure 5:**
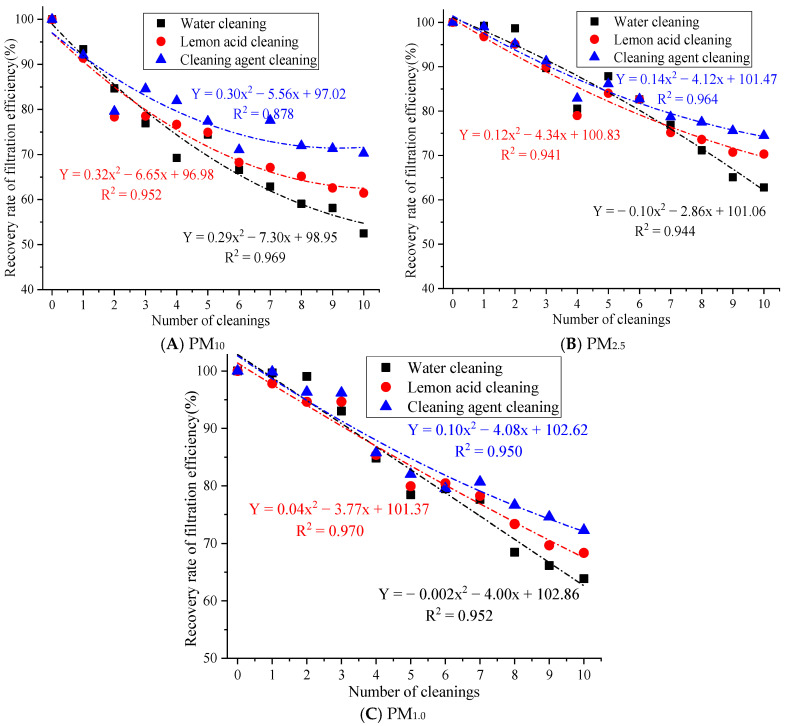
The influence of cleaning times on the filtration efficiency recovery rate.

The filtration efficiency recovery rate after 1 to 10 cleaning cycles is shown in Figure 5.

Figure 5 demonstrates that after 10 cleaning cycles with water, the filtration efficiency recovery rates of PM_10_, PM_2.5_, and PM_1.0_ were 52.48%, 62.80%, and 63.86%, respectively. After 10 cleaning cycles with lemon acid, the filtration efficiency recovery rates of PM_10_, PM_2.5_, and PM_1.0_ were 61.45%, 62.80%, and 63.86%, respectively. After 10 cleaning cycles with the cleaning agent, the filtration efficiency recovery rates for PM_10_, PM_2.5_, and PM_1.0_ were 70.28%, 74.48%, and 72.30%. The filtration efficiency recovery rate of the cleaning agent for PM_10_, PM_2.5_, and PM_1.0_ is above 70%. This result indicates that the synergistic effect of the cleaning solution and ultrasound action is more conducive to restoring the filtration efficiency. This is because ultrasonic cleaning with water may only rely on ultrasonic cavitation to remove loose particles, which results in the limited removal of oil, scale, and heavy metal deposits. At the same time, there are many residual pollutants on the fiber surface, which can easily cause secondary blockages [21]. The lemon acid solution utilizes weak acidity and chelation to remove the scale and metal oxide pollution relatively well, but acidic conditions may cause slight damage to the surface of rGO [25], resulting in the insufficient removal of stubborn organic pollutants. Cleaning agents are generally compound types (surfactant + chelating agent + corrosion inhibitor) [26], which may have the ability to emulsify, chelate, and disperse, which results in the effective removal of composite pollutants, such as oil stains, dust, and microbial films. Therefore, cleaning agents’ cleaning efficiency is significantly better than that of the lemon acid and water.

In addition, the filtration efficiency recovery rate for PM_10_, PM_2.5_, and PM_1.0_ after 10 cleaning cycles with cleaning agents was 17.80%, 11.67%, and 8.44% higher than that of the water cleaning approach and 8.83%, 4.16%, and 3.94% higher than that of the lemon acid method, respectively. Large particles may adhere to the surface, while fine particles are inside the fibers. Cleaning with water can only remove large particles through hydraulic flushing, but this method has no effective stripping ability for fine particles embedded in the pores of the material. Moreover, repeated water flow impacts may easily cause the mechanical deformation of the filter fibers, increasing the porosity of the material and causing uneven pore size distributions [27], resulting in a significant attenuation of the physical screening and inertial collision interception of fine particles.

### 3.4. The Influence of Counting Filtration Efficiency

**Figure 6:**
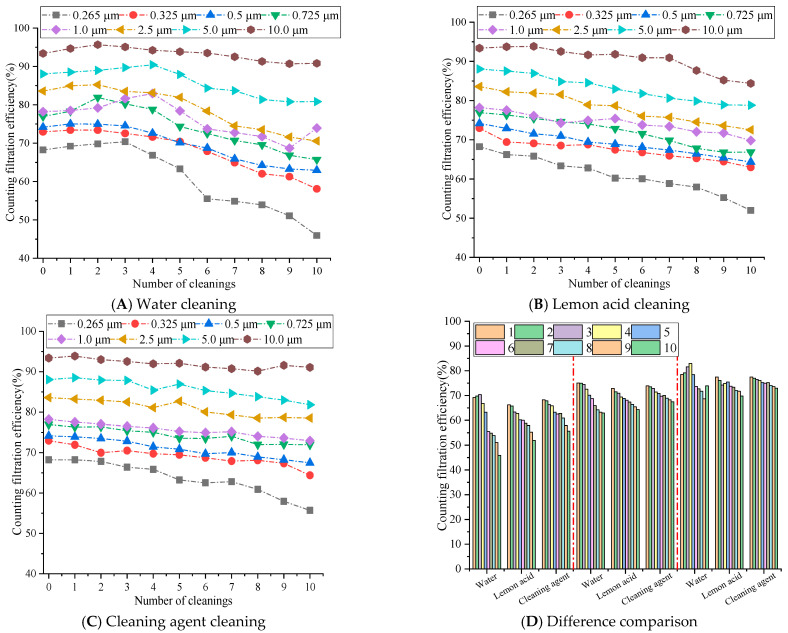
The influence of cleaning times on the counting filtration efficiency.

The counting filtration efficiency differences of rGO filter materials are shown in Figure 6.

Figure 6 demonstrates that the counting filtration efficiency of the rGO filter material for particles of different sizes decreases with the increase in cleaning times, and the efficiency for small particles decreases significantly, more than that of large particles. The water cleaning method exhibited the most significant decrease in efficiency, followed by the lemon acid cleaning approach, while the cleaning agent exhibited the smallest decrease and maintained a relatively high level of efficiency. After 10 cleaning cycles, the filtration efficiency of 10.0 μm particles could still be maintained at over 80%, while the filtration efficiency of 0.265 μm decreased to around 50%. This is because the surface tension of water is high, and the capillary force generated may easily damage the stacked structure of rGO layers [28], resulting in loss of the ability to intercept small-sized particles. This aligns with [21] (Zhang, 2022), which reports water-induced rGO layered structure damage during repeated cleaning. The weak acidity of lemon acid slightly disrupts the hydrogen bonding between rGO layers, resulting in a greater decrease in efficiency compared to cleaning with cleaning agents. Meanwhile, the surface tension of lemon acid is lower than that of water [29], and its capillary damage effect is weaker than that of the pure water cleaning approach [30]. Cleaning agents may reduce the surface tension of liquids and minimize capillary damage during ultrasonic cleaning. At the same time, the adsorption effect of surfactants may clean the pollutants on the surface and pores of rGO membranes without damaging the internal structure [31], achieving a dual effect of cleaning and structural protection. Yet this is also related to its concentration. Relevant papers have demonstrated that when the concentration is too high, it may also corrode the fiber material to a certain extent, causing a significant decrease in filtration efficiency [32].

During 1–5 cleaning cycles, for an average particle size of 0.265 μm, the efficiency of water cleaning on rGO filter materials is 3.00% to 7.04% higher than that of lemon acid methods and 0.05% to 4.00% higher than that of cleaning agents. For average particle size of 0.5 μm, the efficiency of lemon acid cleaning is 1.26% to 3.58% higher, and the efficiency of cleaning agents is 1.10% to 1.68% higher. For average particle size of 1.0 μm, the efficiency of lemon acid cleaning is 0.99% to 8.04% higher, and the efficiency of cleaning agents is 0.99% to 6.84% higher. During 6–10 cleaning cycles, the efficiency differences in each cleaning medium sharply increased, and the cleaning agents exhibited significant long-term stability, while the water cleaning efficiency showed a cliff-like decline. For average particle size of 0.265 μm, the efficiency of cleaning agents on rGO filter materials is 6.86% to 9.77% higher than that of water and 2.48% to 3.99% higher than that of lemon acid cleaning. For average particle size of 0.5 μm, the efficiency of water cleaning is 0.98% to 4.97% higher, and the efficiency of lemon acid cleaning is 1.61% to 3.16% higher. For average particle size of 1.0 μm, the efficiency of water cleaning is 1.23% to 4.93% higher, and the efficiency of lemon acid cleaning is 1.18% to 3.13% higher. Therefore, particle sizes between 0.265 μm and 1.0 μm demonstrate a significant change trend. This is because after more than five cleaning cycles, the capillary pulling effect caused by the high surface tension of water continues to accumulate [33], gradually disrupting internal structure (π-π and hydrogen bonding) [34], leading to an increase in membrane porosity and pore size expansion, resulting in a sharp loss of retention ability for small diameters particles ranging from 0.265 to 1.0 μm. The surfactant molecules of the cleaning agent may adsorb onto the surface of the rGO membrane to form a protective layer, weakening the physical impact of ultrasonic cavitation. The low surface tension significantly may reduce the capillary force of the liquid within the membrane pores [35], avoiding continuous damage to the layered structure. Therefore, after 6–10 cleaning cycles, the filtration efficiency for various sizes particles remains above 80%. Although the weak acidity of lemon acid cleaning may gradually accumulate with the increase in cleaning times, the surface tension of lemon acid is lower than that of pure water, and the capillary damage effect is relatively weak, resulting in a decrease in efficiency between the two [30].

Therefore, water cleaning is only feasible for 1–5 cleaning cycles, while cleaning agents become the optimal medium when multiple cleaning cycles are required for rGO filter materials, due to the minimized physical damage they cause and their long-lasting cleaning ability. Lemon acid can be used as a suboptimal choice for scenarios with moderate cleaning needs.

### 3.5. The Influence of Different Cleaning Solutions on Filtration Resistance

**Figure 7:**
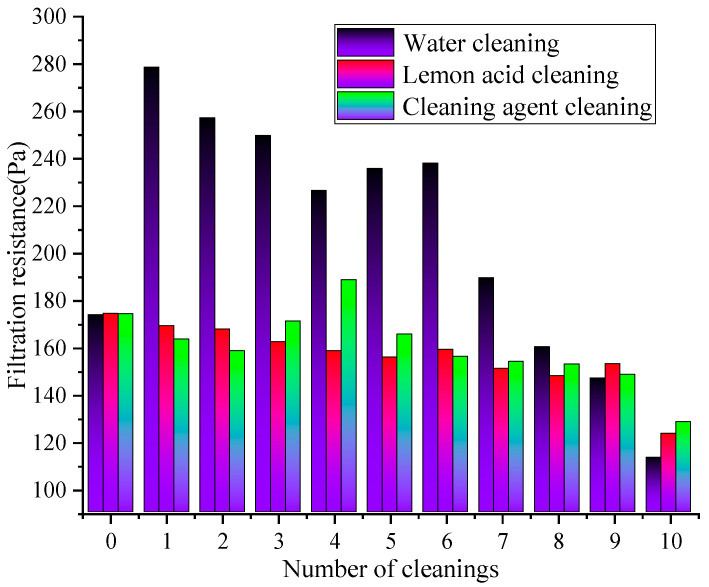
The effect of cleaning times on resistance changes under different cleaning solutions.

The difference in the resistance of the rGO filter material after ultrasonic cleaning using different cleaning solutions is shown in Figure 7.

Figure 7 demonstrates that under the action of different cleaning solutions, the filtration resistance of the rGO filter material continuously decreases with the increase in cleaning times. Under the same cleaning cycles numbers, the resistance after water cleaning is higher than that cleaning agent cleaning and lemon acid cleaning, demonstrating that water cleaning (resistance reduction rate of 34.86%) > lemon acid cleaning (resistance reduction rate of 29.24%) > cleaning agent cleaning (resistance reduction rate of 26.37%). The resistance of the water cleaning remained at a relatively high level, while the cleaning agent cleaning showed the smallest variation with the cleaning cycles numbers, demonstrating the optimal resistance stability. During 1–5 cleaning cycles, the water cleaning method might exhibit the fastest decrease in resistance, followed by the lemon acid, and the cleaning agent approach exhibited the smallest decrease in resistance. During 6–10 cleaning cycles, the rate of the resistance decrease significantly slowed down in all cleaning groups, and the difference between groups widened further. This is because the physical flushing effect of water may quickly remove loose pollutants on the membrane surface, slightly improving pore connectivity, resulting in a rapid decrease in resistance. However, this method cannot remove viscous particles inside the pores. As the rGO layer’s structure continues to be destroyed, the membrane porosity increases while the resistance decreases. The resistance of the lemon acid steadily decreases, and the weak acidic continuous cleaning effect may remove stubborn inorganic pollutants in the membrane. However, the cumulative effect of the structural corrosion may cause the resistance reduction rate to be slower than that of the water cleaning approach [36]. The resistance of the group with the cleaning agent remains the lowest and fluctuates the least. The long-term cleaning and structural protection effects of the surfactant, as well as the formation of an adsorption layer to optimize wetting and reduce air flow friction resistance, result in the rapid stabilization of the resistance after the initial decrease.

### 3.6. The Influence of Different Cleaning Solutions on the Quality Factor

**Figure 8:**
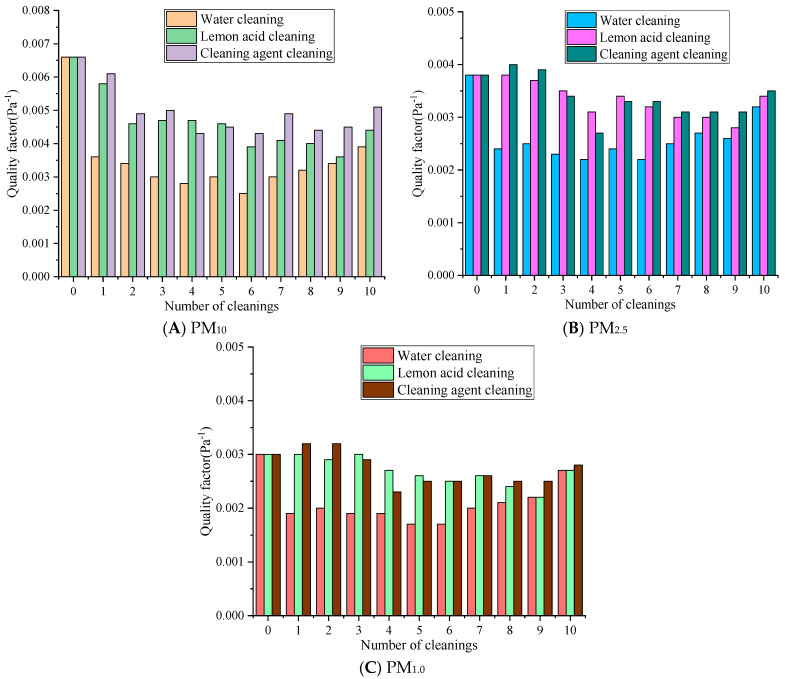
The influence of quality factors with different cleaning solutions.

Figure 8 represents the changes in the quality factor of the rGO material after ultrasonic regeneration with different cleaning solutions.

Figure 8 reveals that with the same number of cleaning cycles, the quality factor values consistently follow the order of cleaning agent cleaning > lemon acid cleaning > water cleaning. For different particle sizes, the quality factor exhibits a pattern of PM_10_ > PM_2.5_ > PM_1.0_, and the quality factor of small-sized particles is more sensitive to the cleaning process. The quality factor of PM_10_ decreased the most in the water cleaning group over 1–5 cleaning cycles, from 0.0066 Pa^−1^ to around 0.0030 Pa^−1^, followed by the lemon acid group at 0.0046 Pa^−1^ and the cleaning agent group at 0.0045 Pa^−1^. Over 6–10 cleaning cycles, the quality factor of the cleaning agent slowly rebounded at first, the lemon acid’s value fluctuated slightly, and the water method’s value remained at a low level. The quality factor of PM_2.5_ decreased from 0.0038 Pa^−1^ to 0.0024 Pa^−1^ in the water cleaning group, to 0.0034 Pa^−1^ in the lemon acid cleaning group, and to 0.0033 Pa^−1^ in the detergent cleaning group over 1–5 cleaning cycles. Over 6–10 cleaning cycles, there was a slight rebound. The quality factor of PM_1.0_ decreased from 0.0030 Pa^−1^ to 0.0017 Pa^−1^, 0.0026 Pa^−1^, and 0.0025 Pa^−1^ in the water, lemon acid, and cleaning agent groups over 1–5 cleaning cycles, respectively. Over 6–10 cleaning cycles, the water cleaning group exhibited relatively significant changes, while the cleaning agent and lemon acid groups were basically the same. Water cleaning may destroy the pore structure and eliminates the electrostatic sites [37], resulting in a sharp decrease in filtration efficiency. At the same time, the resistance reduction associated with water cleaning is limited, so the quality factor value is the lowest, and the decrease is the most severe. The surfactant of the cleaning agent may form a charged adsorption layer, maintaining the electrostatic adsorption of PM_1.0_, so the quality factor can recover in later stages.

Overall, after 10 cleaning cycles, the QF values of PM_10_, PM_2.5_, and PM_1.0_ for the cleaning agent were 0.0012 Pa^−1^ and 0.0007 Pa^−1^, 0.0003 Pa^−1^ and 0.0001 Pa^−1^, 0.0001 Pa^−1^ and 0.0001 Pa^−1^ higher than those in the water and lemon acid groups, respectively. In summary, the synergistic effect of the cleaning agent and ultrasound methods on regeneration is relatively optimal. Lemon acid may be used as a suboptimal choice for pollution scenarios dominated by PM_10_. Water cleaning significantly reduces the quality factor due to its severe damage to the membrane structure and surface characteristics and is only suitable for temporary emergency cleaning operations.

## 4. Conclusions

This study investigated the performances of water, lemon acid, and a cleaning agent on the regeneration of rGO filter materials during ultrasonic cleaning and drew the following conclusions:

1. The synergistic effect of cleaning agents and ultrasound techniques is the optimal regeneration strategy for rGO filter materials. Compared to water and lemon acid, this approach achieves a filtration efficiency for PM_10_, PM_2.5_, PM_1.0_, and maintains filtration efficiency recovery rates above 70% even after 10 cleaning cycles. This confirms that compound cleaning agents can effectively address the core challenge of rGO filter regeneration—balancing pollutant removal with material structure protection.
2. Particle sizes strongly influence regeneration stability: Particles with diameters between 0.265 and 1.0 μm demonstrate the most significant changes in counting filtration efficiency across cleaning cycles. Water cleaning results in the highest filtration resistance and the most severe performance decay, while cleaning agents provide the lowest and most stable resistance. This highlights that the choice of cleaning solution directly impacts the long-term energy efficiency of filtration systems, as a lower resistance reduces operational energy consumption.
3. Cleaning agents outperform water and lemon acid in comprehensive filtration performance (as measured by quality factors, QFs), with sustained advantages after repeated regeneration. Lemon acid exhibits a marginal superiority in PM_2.5_ efficiency for inorganic-rich pollutants but lacks long-term structural stability, while water cleaning causes irreversible damage to rGO’s layered structure and surface functional groups, limiting its practical application to emergency scenarios only.
4. Mechanistically, the water cleaning method relies solely on physical flushing, failing to remove embedded fine particles and damaging rGO’s structure over time. Lemon acid’s weak acidity aids inorganic pollutant removal but induces slight structural corrosion. In contrast, cleaning agents reduce interfacial tension to enhance particle stripping while preserving rGO’s layered structure and functional groups, which enables both efficient regeneration and prolonged material service life.

Conclusions regarding filtration efficiency, resistance, recovery rate, and quality factor are explicitly limited to the selected filtration velocity of 0.8 m/s. Performance at other velocities may vary due to changes in particle capture mechanisms (e.g., inertial impaction, and diffusion) and fluid dynamics, which were not evaluated. Due to equipment constraints, quantitative analysis of residual cleaning agents (e.g., surfactant, corrosion inhibitor) on the rGO surface was not feasible. Qualitative observation via visual inspection (no discoloration or sticky residues) and consistent filtration performance across repeated cycles suggest minimal residue, but this cannot be confirmed quantitatively. The identification of cleaning agents as an optimal medium promotes the green and low-cost application of rGO-based air filters, which is particularly relevant for scenarios requiring long-term, high-efficiency particulate matter control (e.g., industrial dust removal, indoor air purification, and public building ventilation). The lack of oxidation states and mechanical properties of materials after multiple cleaning cycles (such as quality loss and fiber cracking) and SEM, Raman, and XPS methods represent potential areas for future research. As well as the industrial applicability of the proposed materials under high pH conditions and long-term cyclic operation needs to be further verified, since the lack of cyclic durability data cannot fully support their stable performance in practical applications. Conclusions regarding the cleaning agents’ long-term effects on rGO performance are limited to the experimental conditions (three repeated rinses + 60 °C drying). Residual reagents may accumulate over extended cleaning cycles (e.g., >10 cycles) and affect material stability, which requires further verification with residue analysis techniques in future studies. Future research will prioritize systematic durability evaluations, including mass change tracking, morphological observation via electron microscopy, and mechanical strength tests after 10+ cyclic treatments, to comprehensively assess the material stability under harsh conditions. Subsequent studies can focus on the optimization of cleaning solution concentrations, ultrasonic parameter economics, cleaning solution recycling technology, and the effect of surface tension and other parameters on cleaning mechanisms to further advance the industrialization of rGO filter regeneration.

## Figures and Tables

**Table 1:** Calculation information.

Parameter	Specification	Calculation
Effective filter area (A)	0.01 m^2^ (10 cm × 10 cm)	Custom-designed rectangular filter holder; active area defined by sealed perimeter (excluding edge clamping region)
Holder geometry	Rectangular (12 cm × 12 cm outer dimension, 10 cm × 10 cm inner active area)	Made of acrylic resin; 2 cm thick with rubber gaskets for sealing
Sealing/leakage considerations	Rubber O-ring gaskets at filter-medium interface	Leakage test: <0.5% of total flow rate (verified by particle concentration balance before/after holder without filter)
Face velocity (v)	0.8 m/s (optimal, consistent with [22])	Controlled by variable-frequency blower; calibrated via velocity sensor
Flow rate (Q)	28.8 m^3^/h (480 L/min)	Calculation: Q = v × A × 3600 = 0.8 m/s × 0.01 m^2^ ×3600 = 28.8 m^3^/h; converted to L/min: 28,800 L/60 = 480 L/min

## Data Availability

The original contributions presented in this study are included in the article. Further inquiries can be directed to the corresponding authors.
